# News and Coming Events

**Published:** 1907-05-11

**Authors:** 


### NEWS AND COMING EVENTS.

A Congress for the study of Social Hygiene will be held at Lyons on May 13. An exhibition of the latest inventions in the domain of sanitation and municipal hygiene will be opened during the sitting of the Congress.

Dr. G. B. Morgan, J.P., senior surgeon and chairman of the Medical Board of the Sunderland Infirmary, was the recipient of an illuminated address on the completion of 50 years of active service in the interests of the institution. The address, which took the form of a handsomely decorated album enclosed in an ornamented rosewood casket, was presented to Dr. Morgan by Alderman S. Richardson, chairman of committee. Ten years ago, on the completion of 40 years' service, Dr. Morgan was presented with several pieces of silver plate and a ward in the infirmary was named after him.

At a meeting of the Committee of the Eversfield Chest Hospital, St. Leonards-on-Sea, in the offices of the honorary solicitor, Mr. J. Sladen Wing, 1 Delahay Street, Westminster, on the 27th ult., Mr. Edmund Smith Hanbury in the chair, to consider the necessity of increasing the accommodation, enlarging the out-patient department, and considerably widening the existing balconies that more patients may be able to undergo the open-air treatment, the solicitor announced that a client of his had offered £1,000 to help carsy this project into execution. It was proposed to hold a special meeting in the hospital on the 29th inst. to receive plans of this reconstruction from the architects.

The following is the calendar for the present month at the Post-Graduate College, West London Hospital, Hammersmith, W. There will be a lecture or demonstration every day (Saturdays excepted) at 5 p.m.; Dr. A. P. Beddard commences his medical clinical lectures on Wednesday, May 22; Mr. Baldwin lectures on clinical surgery, starting early next month; Major Rogers lectures on tropical medicine, commencing May 13; and Dr. Jones on mental diseases, beginning on Tuesday, May 28. In addition, the following physicians and surgeons attend in the wards at 2.30 p.m. during the present month: Dr. Hood (Thursdays and Mondays), Dr. S. Taylor (Fridays and Tuesdays), Dr. Beddard (Saturdays and Wednesdays), Mr. Keetley (Thursdays), Mr. Edwards (Thursdays), Mr. Paget (Mondays), and Mr. Bidwell (Mondays).

The Royal Free Hospital has recently sustained a great loss owing to the untimely death of Mr. Charles W. Blackman, the Chairman of the Weekly Board, which occurred after a short illness from appendicitis on the 29th ulto. Mr. Blackman had for some years past taken an active part in the administration of the hospital, and he had specially interested himself in the various structural and other improvements which have been carried out during the past few years. On the retirement of Mr. Charles Burt from the Chairmanship of the Weekly Board in June 1905, Mr. Blackman, although the youngest member of the Board, was unanimously voted to that important office by his colleagues, and he had proved himself a most capable chairman. It is difficult in these days for hospitals to secure the services of men in the prime of life, and when they are actively engaged in their own business or profession, but Mr. Blackman took up the work when a comparatively young man, and he freely devoted both his time and energies to the interests of the Royal Free Hospital. The Board have placed on record their high appreciation of the valuable services which Mr. Blackman had rendered to the hospital, and have expressed their sincere sympathy with Mrs. Blackman and the members of her family in their sad bereavement.

Dr. Frederick Taylor, whose connection as physician with Guy's Hospital dates back for 43 years, retired from his post as senior physician to the hospital last week, and has been appointed to the consulting staff.

The annual general meeting of the Medical Defence Union, Limited, will be held at the Midland Hotel, Manchester, on Thursday, May 16, at 4 p.m. Three members of the council—Dr. J. Roche Lynch, Dr. A. A. Mackeith, and Dr. F. J. Wethered, hon. treasurer, who are retiring by ballot under the rules of the Union—are offering themselves for re-election.

A laundry exhibition opens on May 11, at the Agricultural Hall, London, under the auspices of the Society of Laundry Engineers and Allied Trades and the Launderers' Association, with Messrs. Cordingley and Company as business directors. In connection with the event the French laundry associations are organising deputations, which will arrive on Friday and be entertained at a banquet and reception. On the Monday—the second day of the exhibition—delegations from the Dutch and German laundry associations will put in an appearance, thus securing an international character to the event.

Under the immediate patronage of H.R.H. the Princess of Wales, H.R.H. the Princess Royal, His Grace the Duke of Fife, K.T., a Matinée will be given on Thursday, May 16th, at the "Playhouse" Theatre (by kind permission of Mr. Cyril Maude), in aid of "The Girl's Realm" Cot in the Great Ormonde Street Hospital for Children. The following plays will be performed: The Screen Scene from "The School for Scandal," with Miss Winifred Emery, Mr. Fred Terry, Mr. Ben Webster and Mr. Cyril Maude; "The Friend in the Garden," by E. F. Benson; "Compromising Martha," by Keble Howard; and a French Play.

At Southwark Coroner's Court on April 27, Dr. Waldo held an inquiry into the death of John Thomas Harman, aged nine, of Blackwell Street, Walworth, who died under an anæsthetic at Guy's Hospital. The Coroner, before taking evidence, remarked to the jury that the anæsthetic used in this case was ethyl chloride, and since 1895 it had become general as applied to dental operations. He knew of twenty-two fatalities altogether, and in that court he had held four inquests following deaths from the application of this particular anæsthetic during the past three years. The mother of the deceased said that on April 17 he came home from school in the afternoon and complained of pains. The deceased was taken to Guy's Hospital suffering from appendicitis. Christian Hugo Rippman, clinical assistant at Guy's Hospital, said there was administered to deceased three cubic centimetres of ethyl chloride by him in the presence of the house physician. The operation was for removal of tubes and gauze following operation for appendicitis. The case was serious. By the Coroner: Deaths under ethyl chloride were one per 3,000, but there were fewer under ether, and still fewer under chloroform. The reason why ethyl chloride was administered was because it was a short operation. Nitro-oxide gas would not have suited. Robert Davis Colley, house physician, agreed with the preceding witness that three cubic centimetres was a careful dose. He had made a post-mortem examination and found fatty degeneration of the heart and acute pleurisy. There was acute appendicitis. The direct cause of death was poisoning from ethyl chloride, but the poor condition of the child had a serious effect. The jury returned a verdict of "Death by misadventure," and attached no blame to the medical authorities.

